# Amelioration of Morphological Pathology in Cardiac, Respiratory, and Skeletal Muscles Following Intraosseous Administration of Human Dystrophin Expressing Chimeric (DEC) Cells in Duchenne Muscular Dystrophy Model

**DOI:** 10.3390/biomedicines12030586

**Published:** 2024-03-06

**Authors:** Maria Siemionow, Katarzyna Budzynska, Kristina Zalants, Paulina Langa, Sonia Brodowska, Krzysztof Siemionow, Ahlke Heydemann

**Affiliations:** 1 Department of Orthopaedics, University of Illinois at Chicago, Chicago, IL 60607, USA; kat.budzynska@gmail.com (K.B.); kristina.zalants@gmail.com (K.Z.); paulinalanga@gmail.com (P.L.); soniaa.bro@gmail.com (S.B.); siemiok@gmail.com (K.S.); 2 Chair and Department of Traumatology, Orthopaedics, and Surgery of the Hand, Poznan University of Medical Sciences, 61-545 Poznan, Poland; 3 Department of Physiology and Biophysics, University of Illinois at Chicago, Chicago, IL 60607, USA; ahlkeh@uic.edu

**Keywords:** Duchenne Muscular Dystrophy (DMD), Dystrophin Expressing Chimeric (DEC) cells, DEC therapy, systemic-intraosseous administration, cardiac and skeletal muscle morphology, minimal Feret’s fiber diameter, muscle fibrosis, mdx mice, stem cells

## Abstract

Duchenne Muscular Dystrophy (DMD) is a lethal disease caused by mutation in the dystrophin gene. Currently there is no cure for DMD. We introduced a novel human Dystrophin Expressing Chimeric (DEC) cell therapy of myoblast origin and confirmed the safety and efficacy of DEC in the *mdx* mouse models of DMD. In this study, we assessed histological and morphological changes in the cardiac, diaphragm, and gastrocnemius muscles of the *mdx*/*scid* mice after the transplantation of human DEC therapy via the systemic-intraosseous route. The efficacy of different DEC doses was evaluated at 90 days (0.5 × 10^6^ and 1 × 10^6^ DEC cells) and 180 days (1 × 10^6^ and 5 × 10^6^ DEC cells) after administration. The evaluation of Hematoxylin & Eosin (H&E)-stained sectional slices of cardiac, diaphragm, and gastrocnemius muscles included assessment of muscle fiber size by minimal Feret’s diameter method using ImageJ software. The overall improvement in muscle morphology was observed in DMD-affected target muscles in both studies, as evidenced by a shift in fiber size distribution toward the wild type (WT) phenotype and by an increase in the mean Feret’s diameter compared to the vehicle-injected controls. These findings confirm the long-term efficacy of human DEC therapy in the improvement of overall morphological pathology in the muscles affected by DMD and introduce DEC as a novel therapeutic approach for DMD patients.

## 1. Introduction

Duchenne Muscular Dystrophy (DMD) is a lethal, genetic disorder linked to the X chromosome and therefore, it predominantly affects males with an incidence of about 1 in 3500 to 5000 newborns per year [1]. Due to its progressive nature, DMD leads to the gradual deterioration, weakness, and wasting of muscle fibers, resulting in the premature death of DMD patients due to cardiopulmonary complications [2]. DMD is associated with mutations in the dystrophin-encoding gene causing a significant reduction in or lack of dystrophin expression in the sarcomeres. Dystrophin is a large, 420 kDa, structural protein [3] that stabilizes the sarcolemma of the muscle fibers. Due to dystrophin’s absence, muscle fibers become susceptible to contraction-induced damage and undergo the processes of necrosis and repair until the muscle tissue is replaced by fat and fibrous tissue [4]. 

The hallmark of DMD is muscle fiber disarray when compared to the regular shape of healthy muscle fibers [5]. The size of a muscle fiber is determined by fiber growth and maturation, through factors such as exercise and nutrition, whereas diseases such as DMD affect normal muscle growth and have a significant impact on the deterioration of muscle function. In normal muscles, the differences in muscle fiber sizes are minimal; however, in dystrophic muscles significant fiber size variations are observed. Therefore, the DMD-affected muscles of *mdx* mice are more heterogeneous, as indicated by the increased distribution of muscle fiber sizes with diameters below 20 μm and above 70 μm in skeletal muscles [6,7], decreased mean minimal Feret’s diameters of diaphragm myofibers [8], and diffuse variation in cardiac fiber sizes [9]. Another common characteristic of muscle fibers in DMD is a smaller average fiber size when compared to normal muscle tissue [6]. This is due to continued degeneration and an insufficient muscle regeneration pace in the damaged fibers [10,11].

In recent years, a variety of gene- and stem cell (SC)-based studies have been tested for a potential to cure DMD [12,13,14]; however, despite these research efforts, there is still no effective treatment for DMD patients. Recently tested gene therapies, in both preclinical studies and clinical scenarios, include gene editing via viral vectors [15,16], exon skipping, the gene splicing CRISPR/Cas9 (clustered regularly interspaced palindromic repeats) system [17,18], and micro-dystrophin gene delivery via adeno-associated viruses (AAV) [19,20]. The long-term clinical efficacy of these therapies still needs to be determined, and the possible long-term adverse effects of the off-target mutations caused by genetic modified therapies are currently being assessed due to the reported concerns [21]. The alternative approaches include cell-based therapies, which remain among the most hopeful strategies for the treatment and management of muscular dystrophies [14,22]. Stem cell administrations rely on the transplant of either autologous or allogeneic cells [23,24,25,26,27]. Autologous stem cells harvested from DMD-affected patients include mesenchymal stem cells (MSC) and bone-marrow-derived cells, as well as myoblasts, mesoangioblasts, and cardiomyocytes, which undergo ex vivo genetic modifications to deliver functional dystrophin to the muscles affected by the disease [25,27,28,29]. The studies on the allogeneic stem cell transplantation of satellite cells, bone marrow, mesenchymal, muscle-derived stem cells, and induced pluripotent stem cells (iPSC) have demonstrated dystrophin expression in animal models of DMD; however, the major challenges reported by many researchers were either the short-term or limited cell engraftment and allogeneic immune response [30,31,32,33,34]. Despite the application of immunosuppressive treatment, the reports on the long-term maintenance of engraftment are sparse and not consistent [33]. Moreover, the side effects of immunosuppression were and still are of significant concern [26,27,34,35,36]. Thus, to support and maintain long-term engraftment following stem cell transplantation, the development of new, safer therapeutic strategies is essential.

To address these needs, we introduced a concept based on our research testing the tolerogenic effects of donor–recipient chimeric cell (DRCC) therapy created by the ex vivo PEG (polyethylene glycol)-mediated fusion of the hematopoietic cells of bone marrow origin derived from an MHC-mismatched donor and a recipient of vascularized composite allografts (VCA) [37]. This approach resulted in the development of donor-specific chimerism and long-term VCA survival. Encouraged by these outcomes, we applied the same approach of ex vivo PEG-mediated myoblast fusion for the creation of Dystrophin Expressing Chimeric (DEC) cells from normal and DMD-affected donors. We tested this novel therapy in the *mdx* and *mdx*/*scid* mice models of DMD and confirmed long-term DEC cell engraftment, which correlated with a significant increase in dystrophin expression and functional improvements in cardiac, diaphragm, and gastrocnemius muscles confirmed by echocardiography, plethysmography, and standard muscle strength test [28,29,38]. It is clear that the progression of muscle damage in the DMD-affected organs is accompanied by histopathological and morphological changes, specifically in the cardiac and respiratory muscles, leading to cardiopulmonary failure. These still remain the major treatment challenges, despite supportive therapies [39,40]. Thus, it is imperative to assess the effect of current therapies on muscle fiber morphology and the related functional effects. Our studies addressed this issue and confirmed in the preclinical models of DMD the efficacy of DEC therapy as a novel therapeutic strategy for the protection of cardiac, respiratory, and skeletal muscle function, which correlated with reduced fibrosis and inflammation, and improvement in the overall *mdx* morphological pathology.

The goal of this study was to evaluate the efficacy of human DEC therapy in the morphological changes in the DMD-affected muscles, including the cardiac, respiratory, and skeletal muscles, by applying morphological analysis of the minimal Feret’s fiber diameter following injections of different doses of DEC cells via the systemic-intraosseous route. Specifically, we aimed to investigate the degree of heterogeneity of the muscle fiber diameters observed in the *mdx*/*scid* mice model of DMD following systemic-intraosseous administration of human DEC cells. This study summarizes the changes observed in the *mdx* muscle morphology and the related *mdx* pathology, assessed by the minimal Feret’s fiber diameter at two time points of 90 and 180 days after systemic-intraosseous injection of DEC cells, as a measure of DEC therapy efficacy.

We have confirmed the normalization of the overall *mdx* muscle morphology after systemic administration of human DEC therapy, as evidenced by the increase in the muscle fibers’ homogeneity and diameter in the DMD-affected cardiac, respiratory, and skeletal muscles. The results of this study confirm the beneficial effect of DEC on the amelioration of *mdx* pathology and introduce DEC therapy as an alternative option for clinical application in patients suffering from DMD and other muscular dystrophies.

## 2. Materials and Methods

The study was designed to assess the short- (90 days) and long-term (180 days) histological and morphological changes in the muscle fiber diameters after systemic-intraosseous administration of different doses of human DEC therapy to immunodeficient *mdx*/*scid* mice. The same procedures and methodology, including the muscle fiber Feret’s diameter measurements, were performed at the short- and long-term follow-up.

### 2.1. Mice and Animal Care

This study was approved by the Institutional Animal Care and Use Committee (IACUC) of University of Illinois at Chicago, which is approved by the American Association for the Accreditation of Laboratory Animal Care (AAALAC). All animals received humane care in compliance with the ‘Principles of Laboratory Animal Care’ formulated by the National Society for Medical Research and the ‘Guide for the Care and Use of Laboratory Animal Resources’. Six- to eight-week-old male *mdx*/*scid* mice, the animal model for Duchenne Muscular Dystrophy (B10ScSn.Cg-Prkdc*^scid^* Dmd*^mdx^*/J, stock number 018018) with the respective background wild type (WT) mice (C57BL/10ScSnJ, stock number 000476) were purchased from the Jackson Laboratory, Bar Harbor, ME, USA. Animals were kept in a pathogen-free environment on a light/dark cycle. 

### 2.2. Creation of Human DEC Cells

Normal human skeletal myoblasts (MB^N^) were purchased from Lonza Bioscience, cat. CC-2580 (Mapleton, IL, USA) and DMD-affected MBs (MB^DMD^) were purchased from Axol Bioscience Ltd., cat. ax3055 (Little Chesterford, UK) and Creative Bioarray Ltd., cat. CSC-C3604 (Shirley, NY, USA). MBs were cultured in Skeletal Muscle Cell Growth Medium-2 (Lonza Clonetics, cat. CC-3246, Mapleton, IL, USA), supplemented with the human Epidermal Growth Factor (hEGF), 20% Fetal Bovine Serum (FBS), Dexamethasone, and Gentamicin/Amphotericin B (GA) (Lonza Clonetics, cat. CC-4083, Mapleton, IL, USA). Upon reaching 60–70% confluence, MBs were harvested using 0.25% trypsin/EDTA (Sigma/Aldrich, St. Louis, MO, USA). Enzymatic activity was inhibited with 10% serum-supplemented culture media. Human MBs were harvested between passage 3 and 7, which is the optimal passage for the ex vivo cell fusion procedure. Finally, the double-stained (PKH26/PKH67) fused cells were selected via fluorescently activated cell sorting (FACS) (MoFlow Astrios, Beckman Coulter, San Jose, CA, USA) and were used for administration to the femoral bone of *mdx*/*scid* mice. The procedures for creating human DEC cells and the ex vivo PEG-mediated cell fusion were described in detail in our previous papers [28,29,38].

### 2.3. Transplantation of DEC 

Prior to study initiation, age-matched male *mdx*/*scid* mice were ear tagged and randomized into the following experimental groups: vehicle-injected controls (60 µL phosphate-buffered saline—PBS, *n* = 5); DEC therapy group injected with 0.5 × 10^6^ MB^N^/MB^DMD^ cells suspended in 60 µL PBS (*n* = 13); and DEC therapy group injected with 1 × 10^6^ MB^N^/MB^DMD^ DEC cells suspended in 60 µL PBS (*n* = 5). They were observed for 90 days. In a long-term 180 days follow-up, the groups included animals injected with 1 × 10^6^ MB^N^/MB^DMD^ or 5 × 10^6^ MB^N^/MB^DMD^ DEC cells suspended in 60 µL PBS (*n* = 5 per dose) and vehicle-injected controls (*n* = 4). The wild type (WT) normal mice (*n* = 3) were used as a control group for comparative analysis of normal muscle morphology and did not receive any treatment. Before the transplantation, mice were anesthetized with 2% isoflurane in 100% oxygen carrier gas inhalation along with 1 mL/kg buprenorphine subcutaneous injection. A 5 mm incision was made at the lateral mid-thigh level and muscles were separated to expose femoral bone. A tuberculin 25G syringe (cat. ThermoFisher, Waltham, MA, USA) was used for injection of DEC cells in a 60 µL volume of sterile PBS with previous aspiration of bone marrow (60 µL) directly from the femur. Bone wax was applied to the delivery site. Subsequently, muscles were approximated, and the wound was closed using 5-0 nylon sutures. Animals recovered in a heated environment with post-operative monitoring and returned to the colony. The description of intraosseous DEC administration was reported in detail in previous publications [28,29]. 

### 2.4. Histological Analysis

For histological analysis and the assessment of minimal Feret’s fiber diameter, the heart, diaphragm, and gastrocnemius muscle samples were harvested, washed in PBS, fixed in 10% neutral buffered formalin, and embedded in paraffin. Next, the paraffin blocks were cut into 5 μm non-consecutive transverse cross-sections with a distance of 100 µm between the levels. Samples were deparaffinized in xylene and hydrated in a decreasing concentration of ethanol and distilled water. Then, sections were H&E stained according to the manufacturer’s protocol, dehydration was performed via three changes of absolute alcohol (Abcam, ab245880, Cambridge, MA, USA), and they were mounted in a synthetic resin (Poly-Mount, PolySciences Inc., Warrington, PA, USA). A BX51/IX70 Microscope (Olympus, Japan) was used for acquiring images of the stained samples [28,29]. Sections of heart, diaphragm, and gastrocnemius muscles were assessed in 12 regions of interest (ROI) acquired from three non-serial cross-sections from *n* = 3 mice (12 ROI/organ/mouse or 36 ROI/organ/group). Hematoxylin & Eosin (H&E)-stained sections of target organs of DEC-injected *mdx*/*scid* mice were assessed and compared with the WT mice and vehicle-injected controls. To analyze the images of the muscle tissue slides, the ImageJ (1.53a, U.S. National Institute of Health, Bethesda, MD, USA) image analysis software was utilized.

### 2.5. Minimal Feret’s Diameter for Morphological Analysis

**Figure 1:**
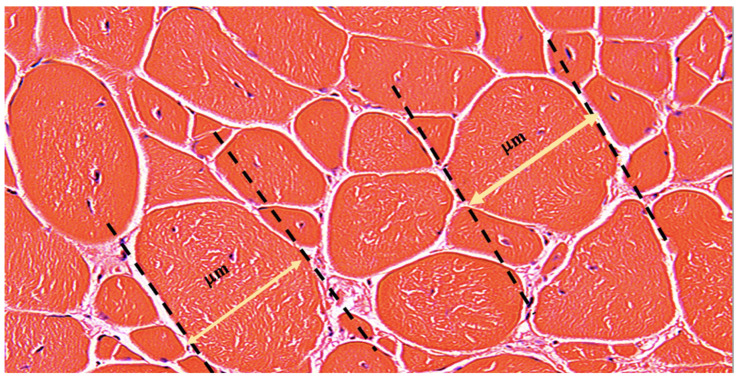
Representative Image of the Minimal Feret’s Fiber Diameter Measurement. The picture represents the H&E-stained transverse section of the Gastrocnemius Muscle (GM) of the 5 × 10^6^ treatment group at 180 days after DEC therapy administration. The yellow double-sided arrow represents the actual minimal Feret’s fiber diameter, which is the shortest distance between two tangents at opposing borders of the muscle fiber. The tangents are drawn for each fiber and diameter is measured in micrometers [μm] after adjusting the scale in the ImageJ software accordingly, as described earlier. Magnification 20×, scale bar 10 μm, Zeiss Meta confocal microscope (Carl Zeiss, Oberkochen, Germany).

Morphological analysis was performed using the minimal Feret’s diameter, a geometrical parameter accounting for the closest possible distance between the two parallel tangents at the opposing border of the muscle fiber [7] (Figure 1). This method provides reliable results as the variance of the fiber size is minimally affected by the angle of sectioning, thereby reducing the measurement errors [7]. The heterogeneity of the muscle fibers was assessed via measurements of the mean minimal Feret’s diameter and the distribution of the fibers falling into three increment ranges, depending on the fiber’s diameter: 0–20 μm, 20–50 μm, and above 50 μm. The number of fibers in each increment range was adjusted relative to the overall quantity of fibers and presented as a percentage value.

### 2.6. Statistical Analysis

The statistical analysis was performed and summarized as means ± SEM (standard error of the mean). To calculate statistical significance, the average values per increment per mouse were used for percentage distribution of myofibers, while individual values were used for calculating the mean minimal Feret’s diameter. GraphPad Prism software (ver. 9.2.1, Dotmatics, Boston, MA, USA) was used for statistical analysis. A two-way ANOVA test was used to define statistical significance. Results were considered significant for *p* < 0.05.

## 3. Results

### 3.1. Local Safety and Tolerance Were Confirmed at 90 and 180 Days Following Transplantation of Human DEC Therapy to the mdx/scid Mice via Systemic-Intraosseous Route

All animals receiving treatment were monitored daily during the first week following the delivery of the DEC cells or PBS. No local side effects, such as hematoma, infection, or edema were observed, and the animals did not show any signs of deterioration of their overall condition. Observations of the animals continued for up to 90 and 180 days post DEC therapy administration. No signs of either immediate or delayed immune reactions were noted throughout the follow-up duration, including anaphylactic reaction, edema, erythema, inflammatory response, or instances of animal mortality.

This study assessed the effect of different doses of DEC therapy on the morphological changes in the DMD-affected muscles, including cardiac, respiratory, and skeletal muscles after systemic-intraosseous injection in *mdx*/*scid* mice. For assessment of the minimal Feret’s diameter, 705 images were acquired and a total number of 64,790 muscle fibers were counted in the respective organs of the heart, diaphragm, and gastrocnemius muscles. The DEC treatment groups and controls are summarized in detail in Table 1 for the 90 days’ study and in Table 2 for the 180 days’ study. 

### 3.2. Transplantation of Human DEC Therapy Results in Improvement of Morphology of Cardiac Muscle at 90 and 180 Days after Intraosseous Transplantation

Cardiomyopathy-induced heart failure is now the primary cause of death in DMD patients. Therefore, we have evaluated the systemic effect of DEC therapy on the morphology and histology of the cardiac muscle by assessment of the minimal Feret’s fiber diameter.

**Figure 2:**
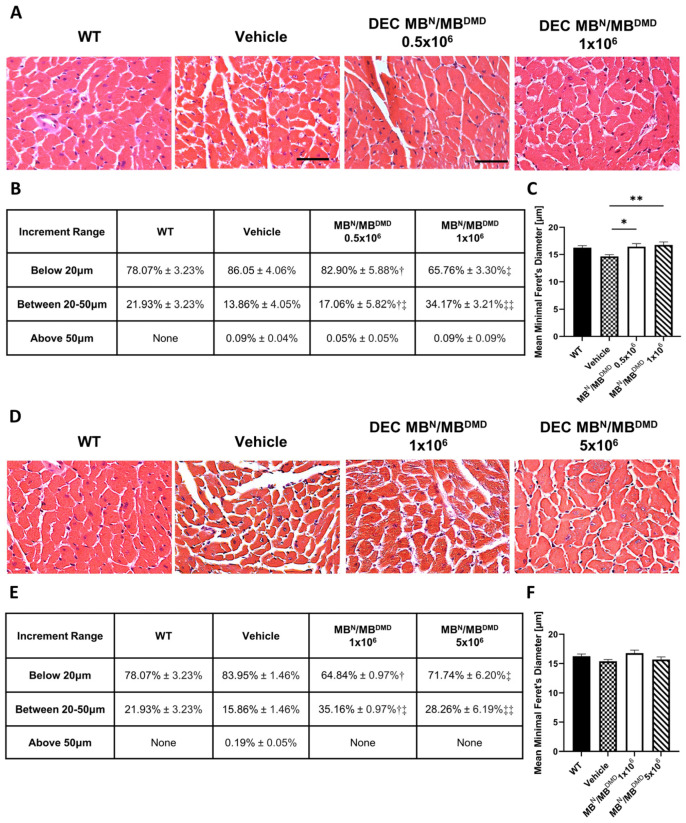
Morphological improvement of the cardiac muscle fibers after 90 and 180 days following the systemic-intraosseous delivery of human DEC therapy. (**A**) Hematoxylin and Eosin (H&E)-stained histological images representing sectional slices of the *mdx*/*scid* mouse heart at 90 days post systemic-intraosseous delivery of DEC cells when compared with the vehicle-injected and wild type (WT) controls. (**B**) The distribution of myofibers based on the minimal Feret’s diameter measurements revealed a dose-dependent effect of DEC therapy on normalization of the cardiac muscle fiber sizes approaching the WT phenotype. † Significant decrease in the percentage of fibers below 20 μm compared to the group injected with 1 × 10^6^ dose of DEC cells (*p* < 0.05); ‡ Significant decrease in the percentage of fibers below 20 μm compared to the group injected with 0.5 × 10^6^ dose of DEC cells (*p* < 0.05) and vehicle-injected controls (*p* < 0.01); †‡ Significant increase in the percentage of fibers within the 20 μm to 50 μm compared to the group injected with 1 × 10^6^ dose of DEC cells (*p* < 0.05); ‡‡ Significant increase in the percentage of fibers within 20 μm to 50 μm compared to the group injected with 0.5 × 10^6^ dose of DEC cells (*p* < 0.05) and vehicle-injected controls (*p* < 0.01). (**C**) Mean minimal Feret’s diameter values revealed a significant increase in fiber size in both DEC-injected groups (0.5 × 10^6^ and 1 × 10^6^) when compared to the vehicle-injected controls. (**D**) Hematoxylin and Eosin (H&E)-stained histological images representing sectional slices of the *mdx*/*scid* mouse heart at 180 days post systemic-intraosseous delivery of DEC cells when compared with the vehicle-injected and wild type (WT) controls. (**E**) The number of fibers based on the minimal Feret’s diameter represents the trend toward the WT phenotype. † Significant decrease in the percentage of fibers below 20 μm compared to the vehicle-injected controls (*p* < 0.001); ‡ Significant decrease in the percentage of fibers below 20 μm compared to the vehicle-injected controls (*p* < 0.05); †‡ Significant increase in the percentage of fibers within 20 μm to 50 μm compared to the vehicle-injected controls (*p* < 0.001); ‡‡ Significant increase in the percentage of fibers within 20 μm to 50 μm compared to the vehicle-injected controls (*p* < 0.05). (**F**) Increase in the mean minimal Feret’s diameter of cardiac muscle fibers in mice treated with both DEC therapy doses (1 × 10^6^ cells and 5 × 10^6^ cells) when compared to the vehicle-injected controls. All data presented as means ± SEM. Two-way ANOVA. * *p* < 0.05, ** *p* < 0.01. For H&E images on (**A**,**D**): magnification 40×, scale bar 50 μm, *n* = 3/group, and 12 ROI/organ/mouse.

Assessment of H&E cross-sections of the heart samples revealed an improvement in the cardiac muscle architecture, characterized by an increase in the overall homogeneity and the fiber size assessed at 90 (Figure 2A) and 180 days (Figure 2D) following intraosseous DEC administration.

At 90 days post-intraosseous DEC transplantation, the myofibers’ distribution was assessed within three tested fiber size increments, and it is summarized as a percentage value in Figure 2B. Evaluation of small fibers revealed a reduction in the percentage of fibers below 20 μm for both doses of DEC cells, when compared with the vehicle controls, accounting for a 3.15% decrease for the 0.5 × 10^6^ dose and a significant 20.29% decrease for the 1 × 10^6^ DEC dose group, thus confirming the dose-dependent effect of DEC therapy. The reduction in the number of small fibers corresponded with an increase in the fiber size range from 20 μm to 50 μm in the DEC-injected mice when compared to the vehicle-injected mice. The results revealed a 3.20% increase for the 0.5 × 10^6^ dose group and a significant 20.31% increase for the 1 × 10^6^ DEC dose group, thus confirming the dose-dependent effect of systemic administration of DEC therapy, bringing the percentage of fiber size distribution closer to that of the WT mice. Finally, there was a negligible number of fibers with a size above 50 μm for both DEC-injected as well as vehicle-injected mice (Figure 2B). 

The mean Feret’s diameter values of cardiac muscle fibers in DEC-injected mice increased significantly after the administration of 0.5 × 10^6^ (16.45 μm ± 0.54 μm) and 1 × 10^6^ (16.76 μm ± 0.53 μm) DEC doses when compared with the vehicle-injected controls (14.64 μm ± 0.34 μm). Interestingly, the assessment of the mean Feret’s diameter in cardiac muscle samples in both DEC therapy groups revealed fiber diameter values comparable to those of the WT mice (16.26 μm ± 0.38 μm) (Figure 2C), further confirming the beneficial effect of DEC therapy and the normalization of cardiac fiber diameters toward the WT phenotype.

At 180 days post-intraosseous DEC transplantation, the myofibers’ distribution was assessed in three tested increment ranges based on minimal Feret’s diameter, and it is summarized as a percentage value in Figure 2E. The number of fibers below 20 μm was significantly reduced in mice injected with both doses of 1 × 10^6^ and 5 × 10^6^ DEC cells, when compared with the vehicle controls, and revealed a decrease of 19.11% and 12.21%, respectively. These findings corresponded with a significant increase of 19.30% (1 × 10^6^) and 12.40% (5 × 10^6^) in the number of larger fibers, falling into the 20 μm to 50 μm increment range, when compared to the vehicle-injected controls. Interestingly, for both DEC doses, there were no fibers falling into an increment range above 50 μm, aligning with the fiber size distribution observed in the WT mice (Figure 2E).

Measurements of mean Feret’s diameter revealed an increase in fiber sizes in both groups injected with DEC therapy when compared with the vehicle controls (15.39 μm ± 0.31 μm). There was a similar effect observed following injection of 1 × 10^6^ DEC cells (16.79 μm ± 0.51 μm) versus 5 × 10^6^ cells (15.69 μm ± 0.45 μm), and this was comparable to the WT mice (16.26 μm ± 0.38 μm) (Figure 2F).

### 3.3. Human DEC Therapy Increases Overall Homogeneity of the Diaphragm at 90 and 180 Days Following Systemic Delivery via Intraosseous Route

Given that respiratory failure is one of the major conditions leading to the early mortality of adolescent boys, we have assessed the effect of human DEC therapy on the amelioration of morphological pathology in the diaphragm muscle.

**Figure 3:**
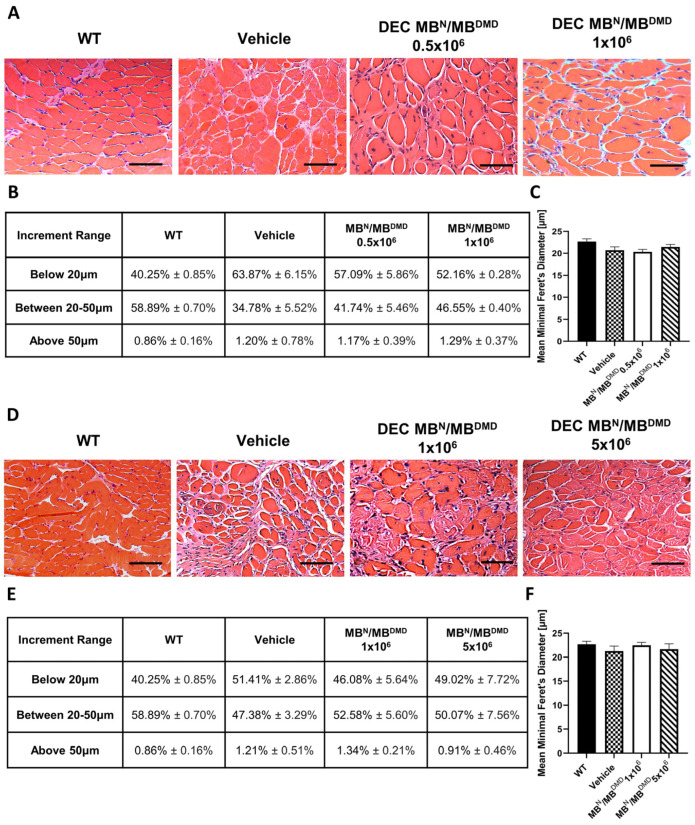
Increase in overall homogeneity of diaphragm after 90 and 180 days following the systemic-intraosseous delivery of human DEC therapy. (**A**) Hematoxylin and Eosin (H&E)-stained histological images representing sectional slices of the *mdx*/*scid* mouse diaphragm at 90 days post systemic-intraosseous delivery of DEC cells when compared with the vehicle-injected and wild type (WT) controls. (**B**) The distribution of fibers falling into three consecutive increment ranges based on minimal Feret’s diameter showed an increase in the percentage of mid-sized fibers in both DEC-injected groups (0.5 × 10^6^ and 1 × 10^6^) with reduction of very small and large fibers when compared with the vehicle-injected controls. (**C**) Measurements of the mean minimal Feret’s diameter revealed an increase in mice injected with 0.5 × 10^6^ comparable to the vehicle-injected controls, and an increase approaching the WT phenotype in mice injected with a higher DEC dose (1 × 10^6^). (**D**) Hematoxylin and Eosin (H&E)-stained histological images representing sectional slices of the *mdx*/*scid* mouse diaphragm at 180 days post systemic-intraosseous delivery of DEC cells when compared with the vehicle-injected and wild type (WT) controls. (**E**) The percentage distribution of myofibers based on the Feret’s diameter showed a decrease in the number of fibers below 20 μm and an increase in the number of larger fibers for the group injected with 1 × 10^6^ dose of DEC cells, with the normalization of diaphragm morphology toward the WT phenotype for mice injected with the dose of 5 × 10^6^ DEC cells, in comparison to the vehicle-injected controls. (**F**) Mean minimal Feret’s diameter values in *mdx*/*scid* mice treated with human DEC therapy revealed no significant increase between both DEC doses and vehicle-injected controls. All data presented as means ± SEM. Two-way ANOVA. For H&E images on (**A**,**D**): magnification 20×, scale bar 100 μm, *n* = 3/ group, and 12 ROI/organ/mouse.

An overall increase in the fiber size and muscle homogeneity could be observed in the histology images of the H&E cross-sections of diaphragm muscle in all DEC-injected groups at both 90 (Figure 3A) and 180 (Figure 3D) days post-administration.

At 90 days following intraosseous DEC transplantation, we evaluated the number of fibers falling into the three increment ranges based on the minimal Feret’s diameter as presented in percentage values in Figure 3B. In the mice that received different doses of DEC cells, the number of fibers below 20 μm was decreased and revealed the following: a 6.78% decrease for the 0.5 × 10^6^ dose and an 11.78% decrease for the 1 × 10^6^ dose, which corresponded with an increase in the percentage of fibers within the 20 μm to 50 μm increment range (6.96% and 11.77%, respectively). This trend indicates a shift in diaphragm fiber distribution aligning with that of the WT mice, confirming a positive effect of intraosseous-systemic DEC administration on the normalization of morphology in the diaphragm muscle. Both DEC-injected and vehicle-injected mice demonstrated an inconsequential count of fibers exceeding 50 μm diameter (Figure 3B).

The mean fiber diameter in mice injected with 0.5 × 10^6^ DEC cells (20.33 μm ± 0.59 μm) was comparable to that of the vehicle-injected controls (20.70 μm ± 0.76 μm). However, administration of a higher DEC dose of 1 × 10^6^ cells revealed an increase in the fiber size (21.45 μm ± 0.56 μm) approaching the WT phenotype (22.68 μm ± 0.61 μm) (Figure 3C).

At 180 days following intraosseous DEC transplantation, the distribution of myofibers based on the fiber diameters was expressed as a percentage value and is summarized in Figure 3E. The number of small fibers decreased by 5.33% in mice injected with 1 × 10^6^ DEC cells and by 2.39% in mice injected with 5 × 10^6^ DEC cells, when compared to the vehicle-injected controls. Furthermore, an increase was observed in the percentage of mid-sized fibers (20 μm to 50 μm) and this accounted for a 5.20% increase for the 1 × 10^6^ DEC dose and a 2.69% increase for the 5 × 10^6^ DEC dose, in comparison to the vehicle-injected controls. Differences between the control and treatment groups in the quantity of fibers ranging above 50 μm were not significant (Figure 3E).

This trend was confirmed by the measurements of the mean minimal Feret’s diameter of diaphragm muscle fibers, where no significant differences were found in fiber diameters between both DEC doses—1 × 10^6^ (22.47 μm ± 0.60 μm) and 5 × 10^6^ (21.66 μm ± 1.31 μm)—and the vehicle-injected controls (21.26 μm ± 1.05 μm) (Figure 3F).

### 3.4. Transplantation of Human DEC Therapy Normalizes Gastrocnemius Muscle Morphology at 90 and 180 Days Following Systemic-Intraosseous Transplantation 

Skeletal muscle strength reduction typically manifests as one of the first signs of the disease in DMD-affected patients. Therefore, to validate the effect of DEC therapy on *mdx* muscle morphology, we have assessed gastrocnemius muscle samples following administration of DEC therapy to the *mdx*/*scid* mice.

**Figure 4:**
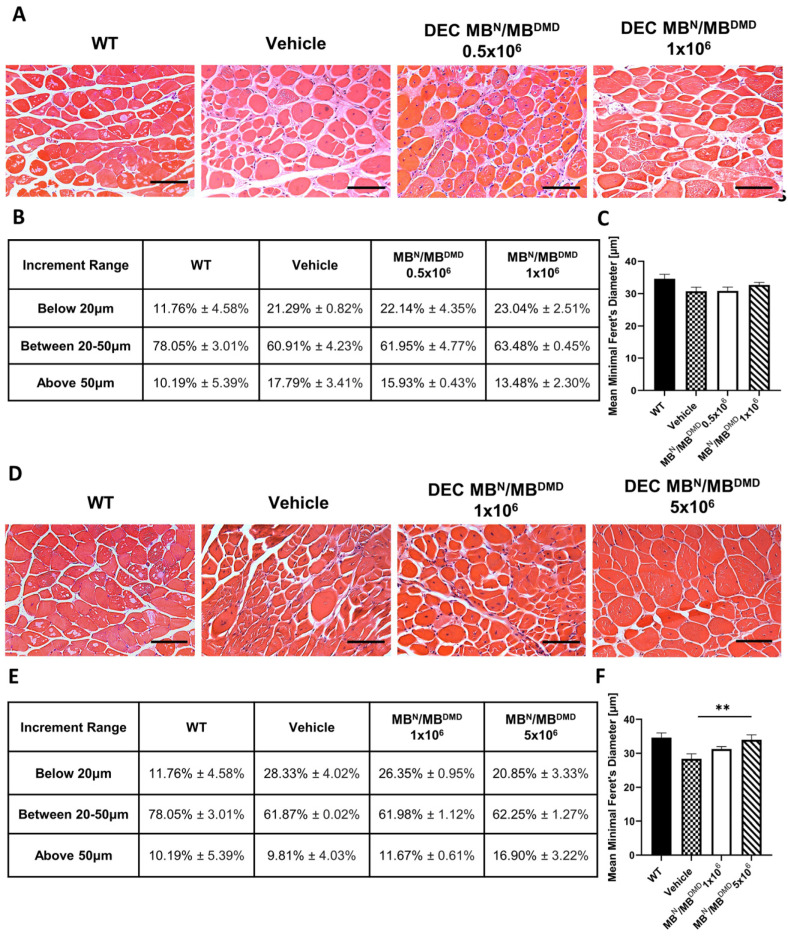
Amelioration of morphological pathology of gastrocnemius muscle after 90 and 180 days following the systemic-intraosseous delivery of human DEC therapy. (**A**) Hematoxylin and Eosin (H&E)-stained histological images representing sectional slices of the *mdx*/*scid* mouse gastrocnemius muscle at 90 days post systemic-intraosseous delivery of DEC cells when compared with the vehicle-injected and wild type (WT) controls. (**B**) An assessment of fiber size distribution determined by the Feret’s diameter revealed an increase in the percentage of small- and mid-sized fibers in both DEC-injected groups (0.5 × 10^6^ and 1 × 10^6^) with reduction in the number of very large fibers (above 50 μm) when compared with the vehicle-injected controls. (**C**) Mean minimal Feret’s diameter of gastrocnemius muscle fibers after systemic DEC transplant confirmed normalization of fiber sizes toward WT phenotype in the group injected with 1 × 10^6^ dose of DEC cells. (**D**) Hematoxylin and Eosin (H&E)-stained histological images representing sectional slices of the *mdx*/*scid* mouse gastrocnemius muscle at 180 days post systemic-intraosseous delivery of DEC cells when compared with the vehicle-injected and wild type (WT) controls. (**E**) The percentage distribution of gastrocnemius muscle fibers depending on the minimal Feret’s diameter revealed a shift toward larger fiber sizes in both treatment groups (1 × 10^6^ and 5 × 10^6^) when compared with the vehicle-injected controls. (**F**) Measurements of the mean minimal Feret’s diameter showed a significant increase in the *mdx*/*scid* mice treated with 5 × 10^6^ dose of DEC therapy when compared with the vehicle-injected controls. All data presented as means ± SEM. Two-way ANOVA. ** *p* < 0.01. For H&E images on A and D: magnification 20×, scale bar 100 μm, *n* = 3/group, and 12 ROI/organ/mouse.

Histological findings in the gastrocnemius muscle samples were similar to those observed in the heart and diaphragm H&E samples. However, the increase in the fiber size diameters between the treatment and control groups was more evident in the 180 days’ study (Figure 4A) compared to the 90 days’ study (Figure 4D).

At 90 days following DEC administration, an assessment of fiber size distribution determined by the fiber diameter was made. This is summarized and presented as a percentage value in Figure 4B. There was an increase in the percentage of fibers falling into the first two increment ranges for both therapy groups, when compared with the vehicle-injected controls. The number of smaller fibers increased marginally by 0.85% and 1.75% for the groups injected with 0.5 × 10^6^ DEC cells and 1 × 10^6^ DEC cells, respectively. Similarly, within the diameter range from 20 μm to 50 μm there was a minor increase of 1.04% in mice injected with 0.5 × 10^6^ DEC dose and of 2.57% in mice injected with 1 × 10^6^ DEC dose. Assessment of the percentage of large fibers, above 50 μm, revealed a desired decrease of 1.86% (0.5 × 10^6^) and 4.31% (1 × 10^6^), when compared to the vehicle-injected mice (Figure 4B). 

Furthermore, the mean minimal Feret’s diameter increased in the group injected with 1 × 10^6^ DEC cells (32.71 μm ± 0.79 μm), when compared to the vehicle-injected controls (30.74 μm ± 1.27 μm), representing the trend toward the WT phenotype (34.66 μm ± 1.38 μm) (Figure 4C). 

At 180 days following DEC transplantation, the number of fibers measuring less than 20 μm, 20 μm to 50 μm, and above 50 μm is summarized and presented as a percentage value in the table in Figure 4E. The number of small fibers was reduced in both treatment groups and revealed a decrease of 1.98% in the 1 × 10^6^ group and a decrease of 7.48% in the 5 × 10^6^ group, when compared with the vehicle-injected controls. There was a minor increase in the percentage of mid-sized fibers, accounting for less than 1% in both DEC-injected groups. However, the number of fibers with a diameter larger than 50 μm increased by 1.87% (1 × 10^6^) and 7.09% (5 × 10^6^), when compared to the vehicle-injected mice, representing the shift toward the larger fiber size in both DEC-injected groups (Figure 4E). 

This trend was also confirmed after the evaluation of the mean minimal Feret’s diameter, which showed an increase in the group of mice injected with 1 × 10^6^ dose of DEC cells (31.28 μm ± 0.70 μm) and a significant increase in the group of mice injected with 5 × 10^6^ dose of DEC cells (34.01 μm ± 1.46 μm), when compared to the vehicle-injected controls (28.40 μm ± 1.44 μm). This confirmed a positive effect of DEC therapy on the normalization of fiber size approaching the WT phenotype (34.66 μm ± 1.38 μm) (Figure 4F).

## 4. Discussion

Duchenne Muscular Dystrophy is one of the most severe types of the inherited muscular dystrophies. It is induced by mutations in the dystrophin gene, leading to the absence or malfunction of the dystrophin protein [1,41]. In the absence of functional dystrophin, myofibers become susceptible to contraction-induced damage, leading to repeated cycles of necrosis and repair, subsequently resulting in the replacement of muscle mass with fat and fibrous tissue [4,42]. The muscles of patients affected by DMD sustain progressive damage, including changes in fiber size and fiber variation, the development of fibrosis and inflammation, the internalization of myonuclei, and alterations in the fiber-type pattern [43]. There are numerous supportive strategies for DMD patients, including the use of corticosteroids, beta-blockers, or angiotensin receptor blockers to target DMD-related cardiomyopathy and to improve patients’ quality of life. However, none improved or halted the advancement of the disease progression [44,45,46]. Therefore, the development of new therapeutic strategies is crucial to address these shortcomings.

Although there is currently no known cure for DMD, several potential therapies have been tested both preclinically and clinically throughout the years. Recent approaches focus on dystrophin restoration through the replacement and/or repair of mutated dystrophin genes or the application of cell-based therapies [47,48]. Different gene therapies designed for enhanced dystrophin expression, including exon skipping, gene editing, and slicing, as well as therapies based on microdystrophin gene delivery via AAVs, have encountered numerous limitations, including adverse immune responses, potential for off-target mutations, and tumorigenicity [15,17,18,19,20,21]. Therefore, concerns have been raised regarding their safety, prompting the exploration of cell-based therapies of myoblast origin as the most promising alternative [25,35]. The initial reports on human myoblast transplantation were encouraging, confirming safety; however, the low efficacy of cell engraftment and the need for immunosuppressive therapy limited their routine application [49]. Multiple approaches and cell lines have been tested, including satellite cells, induced pluripotent stem cells, and the application of mesoangioblasts, cardiomyocytes, allogeneic cardiosphere-derived cells, and MSCs. However, despite the efforts, the achievement of satisfactory therapeutic outcomes has become challenging [13,14,24,25,26,31,32,33]. To address these challenges and meet the current needs, based on our experience in chimeric cell-based therapies and technology [37,50] we have introduced a new myoblast-based chimeric cell line of DEC cells that proved to be safe and efficacious when tested in the *mdx* mice models of DMD [38].

Specifically, to address the problem of myoblast engraftment, we compared different methods of delivering DEC cells, including the local-intramuscular [38] and systemic-intraosseous [28,29] routes. Our findings confirmed the superiority of intraosseous delivery due to its systemic effect on the most severely DMD-affected muscles, including the cardiac, diaphragm, and gastrocnemius muscles. The direct local-intramuscular administration of DEC cells to gastrocnemius muscle demonstrated improvements only in the injected muscle. In contrast, the intraosseous route resulted in systemic effects, confirmed by improvements in cardiac, diaphragm, and gastrocnemius muscle function confirmed by echocardiography, plethysmography, and standard muscle testing [28,29]. 

We have further reported an amelioration in the *mdx* pathological changes, revealed by decreased inflammation and fibrosis as well as the number of centrally nucleated fibers in organs critical for the survival of DMD patients, which correlated with significantly higher values of dystrophin in the heart, diaphragm, and gastrocnemius muscles at 90 [28] and 180 days [29] following systemic-intraosseous DEC administration. We have confirmed the efficacy and protective multi-organ effect of systemic-intraosseous DEC therapy by performing histological, morphological, and immunofluorescent evaluations in the muscles essential for DMD patients’ survival. Furthermore, we assessed the long-term safety and biodistribution of DEC therapy by confirming local and systemic safety at 180 days after intraosseous administration, which led to the first in-human study [51]. 

In the current study, to evaluate the effect of human DEC cells on muscle morphology, we applied an established morphometric method of the minimal Feret’s fiber diameter measurements [6,52] to further test and confirm the efficacy of DEC therapy in the amelioration of morphological abnormalities in the *mdx*-affected muscles.

During the analysis of muscle samples using the Feret’s method, errors are minimized by a geometrical measurement technique in which the actual diameter is measured as the smallest distance between two parallel tangents at opposing borders of the muscle fiber [53]. Moreover, the undeniable advantage of this technique is that it can reduce the impact of different angles of sectioning and human-related errors in measurements while still allowing for sensitive differentiation between dystrophic and non-dystrophic muscle tissues [6,53]. Research reported by Briguet et al. [6] showed that, compared to various parameters like perimeter, area, inner diameter, outer diameter, etc., the minimal Feret’s fiber diameter is the least affected by the orientation of the section plane. In comparison with different methods, such as maximal Feret’s diameter and measuring the shortest or the longest distances between fiber boundaries, the minimal Feret’s fiber diameter technique provided the most precise results with minimized errors [54,55].

Previous studies by Kharraz et al. [4] reported a markedly increased variation in the fiber diameters in DMD-affected muscles, contributing to increased heterogeneity in muscle fiber size. Specifically, the distribution and the abnormal proportion of small and large myofibers are observed to be more abundant in dystrophic muscles [6]. According to the studies by Briquet et al. [6] and Hegarty and Hooper [7], skeletal muscle fiber Feret’s diameters of normal mice usually fall within the range of 20 μm to 70 μm with a minimal number of very small and very large fibers. The fiber size of cardiac muscle is smaller, ranging from 10 to 20 μm [56], while diaphragm fiber size ranges from 20 to 50 μm [57]. Therefore, we anticipated that the systemic-intraosseous administration of human DEC therapy would be associated with a more homogeneous morphology of fiber size. 

To test the effect of DEC therapy on muscle fiber morphology, we applied the Feret’s diameter method of muscle fiber measurements and performed an analysis of a total of 64,790 muscle fibers from the DMD-affected muscles of the heart (20,167 fibers), diaphragm (25,073 fibers), and gastrocnemius muscles (19,550 fibers). The assessment of muscle tissue samples, performed after two different time points at 90 and 180 days following intraosseous transplantation of human DEC cells, revealed a dose-dependent effect on the normalization of the overall homogeneity of myofibers and an increase in muscle fiber diameters. Furthermore, improved muscle morphology correlated with reduced levels of fibrosis and inflammation, confirming improvements in the pathology of *mdx* muscles. These findings are clinically important, since the replacement of muscle mass with fat and fibrous tissue is the main pathophysiological mechanism causing muscle weakness and organ failure leading to premature death of DMD patients [42,58]. It is important to mention that the beneficial effect of DEC therapy was observed in all DMD-affected organs, including heart, diaphragm, and gastrocnemius muscles, and that the long-term efficacy was maintained following administration of a single dose of DEC therapy.

Our study confirmed normalization and an increase in the overall homogeneity of muscle fiber sizes, specifically in the cardiac muscle at 90 days, as well as 180 days after systemic-intraosseous DEC administration. The analysis of the cardiac muscle samples revealed a significant decrease in the number of small size fibers with diameters below 20 μm. Interestingly, this corresponded with a significant increase in the number of fibers with diameters within the 20–50 μm range and this effect was dose dependent. Moreover, both DEC therapy groups revealed normalization of fiber size toward the WT morphology. 

A similar trend was observed in the diaphragm muscle following the injection of two different DEC cell doses, when compared with the vehicle controls. Specifically, the analysis of the distribution of diaphragm muscle fibers revealed an increase in the number of mid-sized fibers and a decrease in the number of very small and very large fibers, approaching the WT phenotype, thus confirming normalization of diaphragm morphology after systemic administration of DEC therapy.

The assessment of gastrocnemius muscle cross-sections at 90 days revealed an increase in the number of fibers falling within the first two increment ranges and a decrease in the number of very large fibers. The most significant effect on the amelioration of morphological pathology was observed in the gastrocnemius muscle at 180 days after administration of the higher DEC dose of 5 × 10^6^ cells. This was confirmed by the shift in the fiber size distribution toward larger fibers and a significant increase in the mean minimal Feret’s diameter values. 

It should be mentioned that this study has some limitations, such as the evaluation of DEC therapy’s effect on the immunodeficient *mdx*/*scid* mice model. While this model allows for the assessment of human cell engraftment and efficacy, it can potentially limit or mask the immune response. Nevertheless, we have previously addressed this limitation and confirmed increased levels of dystrophin expression in immunocompetent *mdx* mice that were directly associated with a significant improvement in muscle function when injected with DEC cells through both intramuscular and intraosseous routes. Importantly, no evidence of side effects was observed, no immune response was observed, and no immunosuppressive therapy was required [38]. Furthermore, the *mdx*/*scid* mouse model of DMD enables testing of DEC therapy based on human cell lines created according to the standard manufacturing protocol based on the fusion of myoblast derived from normal and DMD-affected human donors, and as such represents a clinically relevant model. Moreover, according to Fischer et al. [39], the *mdx* mouse possesses a relatively modest disease, which limits the opportunity to observe progress in the most severe cases. However, despite the natural progression of the disease in the *mdx* aging mice, this study provides clinically relevant and valuable findings, confirming improved muscle fiber morphology and reduced dystrophic phenotype in the DEC-injected mice.

## 5. Conclusions

In this study, the administration of human DEC therapy revealed a positive, dose-dependent effect on the improvement of the morphological pathology in the *mdx*-affected muscles. These findings were confirmed by the normalization of muscle fiber size distribution and an increase in the mean minimal Feret’s diameter of myofibers in cardiac, respiratory, and skeletal muscles at 90 days and 180 days after systemic-intraosseous DEC administration. Moreover, the encouraging results of this study help to support human DEC therapy as a novel and efficient therapeutic approach for DMD patients, which will slow the progress of the disease and have significant impact on their quality of life.

## Figures and Tables

**Table 1:** Summary of the number of images and the number of muscle fibers assessed for the minimal Feret’s diameter at 90 days following transplantation of human DEC therapy in *mdx*/*scid* mice via systemic-intraosseous route.

Organ	Group *	Number of Images	Number of Fibers **
Heart	WT	32	3225
	Vehicle	36	3932
	MB^N^/MB^DMD^ 0.5 × 10^6^	36	3361
	MB^N^/MB^DMD^ 1 × 10^6^	36	3390
Diaphragm	WT	29	4997
	Vehicle	30	4006
	MB^N^/MB^DMD^ 0.5 × 10^6^	36	4706
	MB^N^/MB^DMD^ 1 × 10^6^	35	4445
Gastrocnemius	WT	24	2126
	Vehicle	40	4427
	MB^N^/MB^DMD^ 0.5 × 10^6^	35	3122
	MB^N^/MB^DMD^ 1 × 10^6^	36	3414
Total		405	45,151

* *n* = 3/group, ** 12 ROI/organ/mouse.

**Table 2:** Summary of the number of images and the number of muscle fibers assessed for the minimal Feret’s diameter at 180 days following transplantation of human DEC therapy in *mdx*/*scid* mice via systemic-intraosseous route.

Organ	Group *	Number of Images	Number of Fibers **
Heart	WT	32	3225
	Vehicle	36	2297
	MB^N^/MB^DMD^ 1 × 10^6^	36	2012
	MB^N^/MB^DMD^ 5 × 10^6^	36	1950
Diaphragm	WT	29	4997
	Vehicle	30	2126
	MB^N^/MB^DMD^ 1 × 10^6^	25	2322
	MB^N^/MB^DMD^ 5 × 10^6^	31	2471
Gastrocnemius	WT	24	2126
	Vehicle	34	2120
	MB^N^/MB^DMD^ 1 × 10^6^	36	2219
	MB^N^/MB^DMD^ 5 × 10^6^	36	2122
Total		385	29,987

* *n* = 3 for WT, and *n* = 5 for vehicle, MB^N^/MB^DMD^ 1 × 10^6^, and MB^N^/MB^DMD^ 5 × 10^6^. ** 12 ROI/organ/mouse.

## Data Availability

The data presented in this study are available on request from the corresponding author.
